# Calcium Phosphates for Bone Tissue Regeneration—Influence of Synthesis Method on Physicochemical and Biological Properties

**DOI:** 10.3390/ma18214945

**Published:** 2025-10-29

**Authors:** Julia Sadlik, Edyta Kosińska, Karina Niziołek, Mateusz M. Urbaniak, Agnieszka Sobczak-Kupiec, Dagmara Słota

**Affiliations:** 1 Department of Materials Engineering, Faculty of Materials Engineering and Physics, CUT Doctoral School, Cracow University of Technology, 37 Jana Pawła II Av., 31-864 Kraków, Poland; 2 Faculty of Medicine and Health Science, University of Kalisz, Plac Wojciecha Bogusławskiego 2, 62-800 Kalisz, Poland; 3 Department of Immunology and Infectious Biology, Faculty of Biology and Environmental Protection, University of Lodz, 12/16 Banacha St, 90-237 Łódź, Poland; 4 Department of Materials Engineering, Faculty of Materials Engineering and Physics, Cracow University of Technology, 37 Jana Pawła II Av., 31-864 Kraków, Poland

**Keywords:** calcium phosphates, ceramics, biomaterials, hydroxyapatite, bone

## Abstract

Calcium phosphates, including hydroxyapatite, are widely used biomaterials in bone tissue regeneration due to their bioactivity, osteoconductivity, and similarity to the mineral phase of bone. In this study, various apatite calcium phosphate powders were synthesized using three precipitation methods, with controlled pH conditions and reagent ratios, to assess the effect of the synthesis method on their physicochemical and biological properties. Elemental composition (Ca/P ratio), FT-IR spectroscopy, X-ray diffraction (XRD), scanning electron microscopy (SEM) coupled with EDS, and particle size measurements were used to determine the structure, morphology, and stoichiometry of the obtained powders. The results indicated that the synthesis method and pH significantly affect the phase composition of the material, particle size, and Ca/P ratio, which directly influence their solubility and bioactivity. Microbiological tests, NF-κB transcription factor activation, metabolic activity, and cell compatibility of mouse L929 fibroblasts and human hFOB 1.19 osteoblasts showed good biological tolerance of the obtained powders and no cytotoxic effects. The results confirm that a properly selected synthesis method allows for the control of material properties, which is crucial for applications in bone tissue engineering. The materials show potential for use as bioactive components in bone-related biomaterials.

## 1. Introduction

Nowadays, the problem of an aging population is contributing to the development of fields such as materials engineering and, in particular, biomaterials for use in bone tissue regeneration. Bone, as a mineralized bone tissue, has an irreplaceable role in protecting and supporting the human body. Any defects or injuries to this tissue affect a person’s quality of life [1].

Increasing age also leads to an increased number of skeletal surgeries. One of the most challenging cases is craniofacial surgery, which often requires a personalized approach. The repair of deformed facial bones is made possible by customized 3D-printed implants, and the materials from which these implants are fabricated play an important role in their performance. Currently, a wide range of materials are used for bone reconstruction, from those of natural origin to synthetic ones. These include metallic, polymeric, and ceramic materials. An increasingly common solution is the use of composite systems, and in the case of 3D printing technology, ceramic-polymer granulates or pellets. The ceramic phase in this case can be based on calcium phosphates.

Calcium phosphates (CaPs) are among the most promising biomaterials, formed by the reaction between calcium and phosphorus ions. Their main advantage is that they constitute the mineral component of bone and teeth [2]. Typical examples of minerals belonging to CaPs are hydroxyapatite (HAp), tricalcium phosphate (TCP), and biphasic calcium phosphate (BCP) [1]. They have been proven to integrate with the surrounding bone tissue, which positively influences bone regeneration. Currently, calcium phosphate-based materials are an excellent alternative to bone grafts [3]. They are widely used, especially as dental implants or in maxillofacial surgery [3,4,5,6,7].

Biomaterials must meet a number of requirements to be safely used in the human body. One of the most important features that a material for implant applications must possess is non-toxicity. Achieving this goal is often accomplished through careful synthesis, design, and modification of biomaterials. All steps along the route of material manufacturing must be selected so that the material is ultimately biocompatible [8]. Another important property is the appropriate surface roughness, depending on the application. It provides an important function when forming a bond between the material and bone tissue [9]. The combination of properties such as porosity and surface texture is essential during bone growth and osteointegration [10,11,12].

A problem that often arises when designing biomaterials is stress shielding. The main problem occurs when there is a difference in the Young’s modulus of the bone and the implant material. This results in bone resorption. Many materials have too high a Young’s modulus compared to bone tissue [13]. For this reason, it is becoming increasingly popular to combine two types of materials to create a composite with new and improved properties. Another way to control the Young’s modulus is to manipulate the porosity of the biomaterial [14,15,16].

One of the most popular calcium phosphates used in medicine and many health applications is hydroxyapatite. It is particularly used in bone repair due to its high calcium content. It is the most abundant crystalline phase of biomaterials in human bones and accounts for about 70% of the dry weight of bone tissue [17]. This compound is usually represented by the chemical structure Ca_10_(PO_4_)_6_(OH)_2_ to indicate the hexagonal unit target of HAp [18].

Hydroxyapatite can be synthesized in two ways: by means of a natural process and via artificial synthesis. The hexagonal structure of naturally produced HAp can contain defects that are replaced by other ions or vacancies. On the other hand, defects that can appear in synthesized HAp usually depend on the manufacturing procedure, including synthesis conditions [19,20]. Although it demonstrates good chemical stability, its mechanical properties are poor. However, characteristics such as the coefficient of friction, wear resistance, and hardness of dense HAp are similar to the values of natural mineralized tissues. Due to its low fracture toughness, it is not used as a load-bearing implant. Improving these aspects is possible by designing composites with a polymer. This is especially important for materials for bone tissue regeneration applications [21,22,23,24]. Stoichiometric HAp has a Ca/P ratio of 1.67 and is seen as osteoconductive but not osteoinductive. Improving these properties is possible by combining it with other materials or via ionic substitution. There is a way to improve these properties by using ionic substitutions. Anionic substitutions have been found to improve the bioactivity of hydroxyapatite, while cationic substitutions with Mg^2+^ magnesium ions can result in better biological properties [25,26]. Another common type of calcium phosphate is TCP. We can distinguish between two crystalline phases, α-TCP and β-TCP. The term TCP is used for a phase with a chemical composition of Ca_3_(PO_4_)_2_ and a Ca/P ratio of 1.5 [27,28]. TCP shows good stability and can be stored in a dry environment at room temperature for a long period of time. It should be added that in considering α-TCP and β-TCP, the more stable phase is β-TCP [19]. In medical applications, β-TCP shows better osteoconductivity and osteoinductivity compared to hydroxyapatite, so it is often the one used in bone cements and bioceramics [29,30]. The resorption rate of pure α -TCP has been found to be higher than the formation of new bone tissue, leading to an imbalance between these processes. For this reason, this phase is used in combination with other calcium phosphates. A better resorption rate is possessed by β-TCP, which has greater potential in bone regeneration applications. Moreover, compared to HAp, β-TCP has better biodegradability and a better resporption rate, which positively affects the biocompatibility of the material [7,22]. Another type of calcium phosphates we can distinguish are amorphous calcium phosphates (ACPs). They represent a special form of CaP with variable chemical compositions. They are characterized by a lack of long-range order. They lack well-defined stoichiometry. ACPs exhibit a fairly wide range of Ca/P molar ratios, from about 1.15 to 1.67, depending on synthesis conditions [31,32]. Developments in fields such as materials engineering, biology, technology, and their combination make synthetic CaPs promising biomaterials. They provide similar bone conditions that promote cell proliferation and differentiation and thus further tissue regeneration. They are characterized by good biocompatibility and exhibit osteoconductive and osteoinductive properties [1,20].

The purpose of the present study was to synthesize calcium phosphate powders with different Ca/P ratios using different synthesis methods and to determine their physicochemical and biological properties.

## 2. Materials and Methods

### 2.1. Materials

Ceramic was synthesized using phosphoric acid (H_3_PO_4_) from POCH Basic (Gliwice, Poland) (CAS no. 7697-37-2), calcium hydroxide (Ca(OH)_2_ from Chempur (Piekary Ślaskie, Poland) (CAS no. 1305-62-0), and ammonia water (NH_4_OH, 25%) (CAS no. 1336-21-6) from STANLAB (Lublin, Poland); also, calcium acetate monohydrate (Ca(CH_3_CO_2_)_2_·H_2_O) and sodium phosphate dibasic (Na_2_HPO_4_) were obtained from Sigma-Aldrich (Darmstadt, Germany), ammonium dihydrogen phosphate (NH_4_H_2_PO_4_) was obtained from Chempur (Piekary Śląskie, Poland) (CAS no. 7722-76-1), and calcium nitrate tetrahydrate (Ca(NO_3_)_2_·4H_2_O) was obtained from Chempur (Piekary Śląskie, Poland) (CAS no. 13477-34-4).

The following reagents were used for the biological tests: Roswell Park Memorial Institute (RPMI)-1640 medium, penicillin (100 U/mL), streptomycin (100 µg/mL) (CAS no. 57-92-1), lipopolysaccharide (LPS) from *Escherichia coli* O55:B5, and dimethyl sulfoxide (DMSO) (CAS no. 67-68-5) were purchased from Sigma Aldrich, Darmstadt, Germany. 3-(4,5-dimethylthiazol-2-yl)-2,5-diphenyltetrazolium bromide (MTT) was obtained from Sigma Aldrich (CAS no. 298-93-1), Saint Louis, MO, USA. DMEM/F12 medium and geneticin (CAS no. 108321-42-2) were purchased from Gibco, Zug, Switzerland. Selective agents, normocin (100 μg/mL) and blasticidin (10 μg/mL) (CAS no. 3513-03-9), as well as QUANTI-Blue™, were obtained from InvivoGen, San Diego, CA, USA.

### 2.2. Preparation of Powders

Ceramic powders were synthesized using three wet precipitation methods that differed in the reactants employed. Each synthesis required precise pH control of the reaction medium, achieved through the addition of 25% ammonia water.

*Acid–Base Precipitation Method (ABP)*

Ceramic powders were synthesized via the acid–base precipitation method using 500 mL of 0.3 M orthophosphoric acid (H_3_PO_4_) and 500 mL of calcium hydroxide (Ca(OH)_2_) suspension. The amount of Ca(OH)_2_ was adjusted according to the desired Ca/P molar ratios in the final products, which were 1.0 for ABP 1, 1.5 for ABP 2, and 1.67 for ABP 3.

During synthesis, the phosphoric acid solution was added dropwise to the calcium hydroxide suspension at a rate of approximately one drop per second under continuous magnetic stirring (500 rpm) at room temperature. The pH of the reaction medium was carefully controlled, with it being maintained at approximately 6 for ABP 1, 7 for ABP 2, and 11 for ABP 3, to promote the formation of distinct calcium phosphate phases.

*Ammonium Nitrate Precipitation Method (ANP)*

This method involved dropping 0.36 M (NH_4_H_2_PO_4_) at a rate of one drop per second into a 0.6 M solution of (Ca(NO_3_)_2_·4H_2_O). The entire synthesis was carried out at room temperature under conditions of vigorous magnetic stirring (500 rpm), with pH maintained under conditions analogous to those described above. Three powders, labelled ANP 1, ANP 2, and ANP 3, were obtained, differing in the amount of Ca(NO_3_)_2_·4H_2_O solution used—120 mL, 180 mL, and 200 mL, respectively.

*Acetate Precipitation Method (ACP)*

Ceramic powders obtained using this method were synthesized at the boiling point of the reagents. The main reactants, used in the specified ratio, were 0.32 M (Na_2_HPO_4_) and 0.128 M ((CH_3_COO)_2_Ca). The synthesis was carried out in a three-necked flask containing the Na_2_HPO_4_ solution, with the pH of the reaction controlled using 25% ammonia water—maintained at approximately pH 6 for ACP 1, pH 7 for APC 2, and pH 11 for ACP 3. Subsequently, the (CH_3_COO)_2_Ca solution was added dropwise into the flask at a rate of one drop per second. Three powders, ACP 1, ACP 2, and ACP 3, were obtained, differing in the amount of (CH_3_COO)_2_Ca solution used—120 mL, 180 mL, and 200 mL, respectively.

For all synthesis routes, the post-synthesis procedure was identical: after complete addition of the reactants, the resulting suspension was stirred for 1 h, allowed to settle for 24 h, and subsequently washed with distilled water until a neutral pH was reached. The suspensions were freeze-dried at −50 °C for 48 h using a Martin Christ Alpha 1–2 LDplus freeze dryer (Osterode am Harz, Germany), and the obtained ceramic powders were gently ground in an agate mortar to remove soft agglomerates.

### 2.3. Determination of Calcium and Phosphorus Content

In order to determine the calcium content of the materials, a procedure was performed according to PN-R-64803 [33]. The method involves the dissolution of the sample, complexation of calcium ions, and subsequent titration using a complexometric technique. The analysis was performed in triplicate, and the results were used for further calculations according to Equation (1):

(1) $$\%Ca=\frac{0.04008\cdot V_{1}\cdot c\cdot V_{2}}{m\cdot V_{3}}\cdot 100\%$$

*V*_1_—*volume of EDTA solution used during titration [mL];*

*c*—*titer of EDTA solution [mol/L];*

*V*_2_—*volume of the solution of the test sample [mL];*

*V*_3_—*volume of solution taken for titration [mL];*

*m*—*weight of ceramic sample [g].*

For the determination of phosphorus content, a procedure was carried out according to PN 80/C 87015 [34]. The procedure involved sample mineralization, dilution, and filtration, followed by preparation of a measuring solution. Measurement was carried out using a GENESYS 180 UV-Vis spectrophotometer from Thermo Scientific (Loughborough, UK). Each material was tested in triplicate and the data obtained were entered into Equation (2):

(2) $$\%P=\frac{\left(\frac{M_{1}\cdot V_{1}\cdot 100\%}{m\cdot V_{2}\cdot 1000}\right)}{2.29}$$

*M*_1_*—P_2_O_5_ content in the analyzed sample determined from the standard curve* *[mg/mL];*

*V*_1_ *—the volume of the volumetric flask used for phosphorus extraction [mL];*

*V*_2_*—volume of solution taken for analysis*;

2.29—*conversion factor from P_2_O_5_ to P [mL];*

*m—weight of ceramic sample [g].*

### 2.4. Fourier-Transform Infrared Spectroscopy Analysis

Fourier-transform infrared spectroscopy (FT-IR) was used to determine the functional groups present in the synthesized ceramic–calcium phosphate powders. The analysis also aimed to observe possible differences resulting from the powder production and the Ca/P molar ratios used. The study was carried out using a Nicolet iS5 91 FT-IR spectrometer equipped with an iD7 ATR attachment (Thermo Scientific, Loughborough, UK). The measurements were performed in the range of 4000–400 cm^−1^ and a resolution of 4.0 cm^−1^ at room temperature.

### 2.5. X-Ray Diffraction Analysis

The synthesized ceramic powders were structurally characterized using X-ray diffraction (XRD). The measurements were performed using a Malvern Panalytical Aeris X-ray diffractometer, equipped with a PIXcel1D-Medipix3 detector (Malvern, UK) and Cu Kα radiation. Data were collected over a 2θ range of 10° to 100°, with a step size of 0.0027166° and a dwell time of 340.425 s per step, ensuring high-resolution results.

### 2.6. Particle Size Analysis

The particle size distribution of the ceramic powders was determined using an Anton-Paar PSA 1190LD laser particle size analyzer (Anton Paar GmbH, Graz, Austria). Distilled water served as the dispersing medium. The device operated over a measurement range of 0.04 µm to 2500 µm. Before analysis, the powders were finely ground using a ceramic mortar. Three measurements were taken for each sample, and the average values were calculated using Kalliope Professional software (version 2.22.1, Anton Paar GmbH, Graz, Austria). The results were then presented as average particle size distribution curves.

### 2.7. Morphology Analysis

The surface morphology and grain shape of the synthesized powders were analyzed using a JEOL IT200 scanning electron microscope (SEM) (JEOL Ltd., Peabody, MA, USA) equipped with an energy-dispersive spectroscopy (EDS) detector. Following surface imaging, EDS spectra and elemental mapping were conducted. Prior to SEM examination, the samples were coated with a thin gold layer using a DII-29030SCTR Smart Coater sputtering device (JEOL Ltd., MA, USA) to enhance conductivity.

### 2.8. Sterilization of Ceramic Powders

Prior to the biological experiments, all ceramic powders (ABP, ANP, and ACP) were sterilized by means of gamma irradiation (35 kGy gamma rays; ^60^Co source) at the Institute of Applied Radiation Chemistry, Lodz University of Technology (Lodz, Poland), as previously described [35].

### 2.9. Cell Cultures

The L929 (CCL-1) reference mouse skin fibroblasts were obtained from the American Type Culture Collection (ATCC, Manassas, VA, USA). Prior to experiments, fibroblasts were cultured in Roswell Park Memorial Institute (RPMI)-1640 medium supplemented with heat-inactivated fetal bovine serum (10%; FBS; PAN-Biotech GmbH, Aidenbach, Germany), penicillin (100 U/mL), and streptomycin (100 µg/mL) (Sigma Aldrich, Darmstadt, Germany) at a temperature of 37 °C in a humidified air atmosphere containing 5% CO_2_ [36].

The THP1-Blue NF-κB human monocytes (InvivoGen, San Diego, CA, USA) were cultured in selective RPMI-1640 medium supplemented with heat-inactivated FBS (10%), 100 U/mL penicillin, 100 μg/mL streptomycin, 2 mM glutamine, and selective agents (100 μg/mL normocin and 10 μg/mL blasticidin) in a humidified 5% CO_2_ atmosphere at 37 °C [36].

The hFOB 1.19 human osteoblasts (CRL-3602, ATCC, Manassas, VA, USA) were cultured in Dulbecco’s Modified Eagle’s Medium/Ham’s Nutrient Mixture F12 without phenol red (DMEM/F12, Gibco, Zug, Switzerland) with the addition of heat-inactivated FBS (10%) and geneticin (0.3 mg/mL; Gibco, Zug, Switzerland) in the conditions of a cell culture incubator (33 °C, 5% CO_2_) [37].

Cell viability and density were established using a Bürker chamber (Blaubrand, Wertheim, Germany) and trypan blue exclusion assay, respectively. Cell suspensions with viability exceeding 95% were used in cytocompatibility experiments. Cell culture morphology was monitored using an inverted microscope (Motic AE2000, Xiamen, China) [36,37].

### 2.10. Cytocompatibility

The metabolic activity and cytocompatibility of reference mouse L929 fibroblasts and human hFOB 1.19 osteoblasts exposed to the tested sterile ceramic powders (ABP, ANP, and ACP) at concentrations of 0.1 mg/mL, 0.5 mg/mL, and 1 mg/mL were evaluated using a 3-(4,5-dimethylthiazol-2-yl)-2,5-diphenyltetrazolium bromide (MTT, Sigma Aldrich, Saint Louis, MO, USA) reduction assay, as recommended by ISO 10993-5:2009 [38] and as described previously [36,37,39]. Next, 2 × 10^5^ L929 or 4 × 10^5^ hFOB 1.19 cells/mL were transferred (100 µL/well) into 96-well culture plates and incubated overnight (37 °C for L929 cells and 33 °C for hFOB 1.19 cells, 5% CO_2_) to recreate cell monolayers. Briefly, after 24 h incubation of the cells with the tested ceramic powders, 20 µL of MTT (5 mg/mL) was added to each well, and incubation was continued for the next 4 h. In the next step, the plates were centrifuged (1200 rpm, 10 min), and the supernatants were removed and replaced with 200 µL of DMSO (dimethyl sulfoxide). The absorbance was measured at 570 nm using a Multiskan EX reader (Thermo Scientific, Waltham, MA, USA). Cell cultures in a medium without the tested ceramic powders were used as a non-treated (NTC) control of metabolic activity.

### 2.11. NF-κB Activation

THP1-Blue™ NF-κB monocytes (InvivoGen, San Diego, CA, USA) were used to determine the activation of the NF-κB signal transduction pathway, as previously described [36]. In this in vitro model, the induction of NF-κB results in the secretion of embryonic alkaline phosphatase (SEAP) by THP1-Blue™ NF-κB monocytes. Freshly prepared suspensions of monocytes in selective RPMI-1640 medium were distributed to the wells of culture plates (1 × 10^5^ cells/well, 100 μL). Then, 100 µL of twofold-concentrated ceramic powders (ABP, ANP, and ACP) were added to selected wells to a final concentration of 0.1 mg/mL, 0.5 mg/mL, and 1 mg/mL. Monocytes were incubated for 24 h in a humidified 5% CO_2_ atmosphere of a cell incubator at 37 °C. Cells in selective RPMI-1640 alone served as a non-treated (NTC) control, whereas monocytes stimulated with 10 ng/mL LPS from *Escherichia coli* O55:B5 (Sigma Aldrich, Darmstadt, Germany) were used as a treated (TC) control. The level of SEAP secretion was determined spectrophotometrically in the cell culture supernatants. Cell-free supernatant (20 µL) was mixed with 180 µL QUANTI-Blue™ (InvivoGen, San Diego, CA, USA) and incubated at 37 °C for 4 h. Absorbance was measured at 620 nm using a Multiskan EX reader (Thermo Scientific, Waltham, MA, USA).

### 2.12. Statistical Analysis

The Kolmogorov–Smirnov test was used to test the normality of the data. Intergroup outcomes were compared for statistical significance using ANOVA (analysis of variance) followed by Dunnett’s post hoc test. The differences were considered significant at the *p*-value < 0.05. All analyses were performed using GraphPad Prism 9 software (GraphPad Software, San Diego, CA, USA). The experiments were performed at least five times.

## 3. Results

### 3.1. Determination of Calcium and Phosphorus Content Analysis

When analyzing the obtained Ca/P molar ratio results for bioceramic powders, they should be compared to the theoretical values for the main calcium phosphates used in biomaterials. According to literature data, HAp is characterized by a Ca/P ratio of 1.67, while other phosphates, such as TCP, have lower values (Ca/P = 1.5 for β-TCP; Ca/P = 1.5 for α-TCP) [40,41]. This tells us that there is a wide range of apatites with different Ca/P ratios commonly used. The results obtained indicate that both the synthesis method used and the controlled parameters (mainly pH and reagent ratio) significantly affect the final Ca/P ratio (Table 1).

In the case of the acid–base precipitation (ABP) method, a gradual increase in the Ca/P ratio was observed with increasing pH, from 1.43 for pH = 6 (ABP 1), through 1.55 for pH = 7 (ABP 2), to 1.76 for pH = 11 (ABP 3). A similar relationship was observed for the ammonium nitrate (ANP) method, where the Ca/P ratio increased from 1.32 for ANP 1 to 1.88 for ANP 3. The acetate method (ACP) showed slightly less sensitivity to pH but also revealed an increase in the Ca/P ratio from 1.42 to 1.72. Ca/P values close to 1.67 were obtained for samples ABP 2 (1.55), ANP 2 (1.63), and ACP 2 (1.60), which indicates the possibility of obtaining materials with a composition close to stoichiometric hydroxyapatite with the appropriate selection of parameters. In contrast, samples ABP 3 (1.76), ANP 3 (1.88), and ACP 3 (1.72) showed a Ca/P ratio higher than 1.67, which may indicate the presence of calcium-rich phases or an excess of Ca(OH)_2_ remaining after synthesis. Such excess calcium, although potentially increasing the bioactivity of the material (due to the possibility of faster release of Ca^2+^ ions), may also affect its phase stability and solubility in vivo.

### 3.2. Fourier-Transform Infrared Spectroscopy Analysis

**Figure 1:**
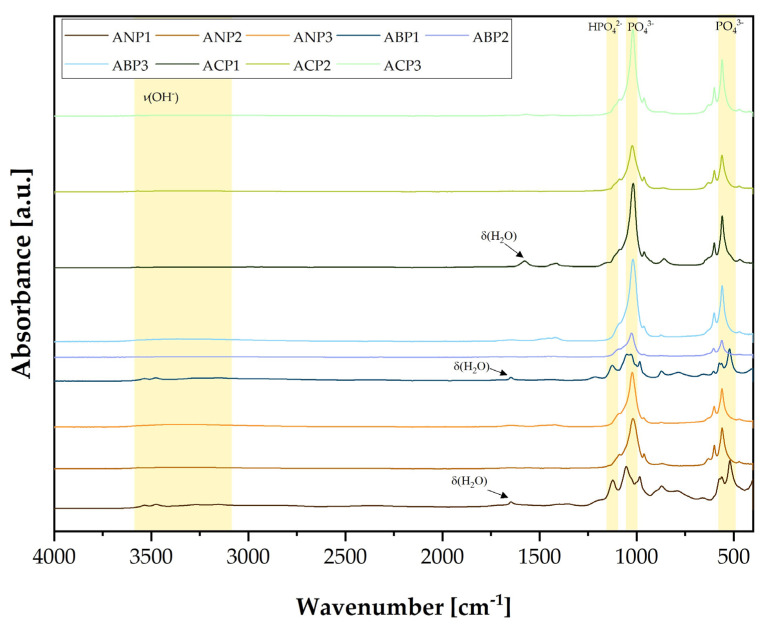
FT-IR spectra of calcium phosphates obtained using different synthesis methods.

Figure 1 presents the FT-IR spectra of the produced calcium phosphates using different methods and with different Ca/P molar ratios. The fabricated ceramic powders were grouped according to the fabrication methods. The purple colors and the abbreviation ANP correspond to ceramic powders that were produced using the ammonium method, the pink colors (ABP) correspond to calcium phosphate ceramics obtained using the acid–base method, and the green shades (ACP) correspond to powders synthesized using the acetate method. Three ceramic powders differing in Ca/P ratio were produced using each method. Ceramic powders with the smallest Ca/P ratio, especially those produced using the ammonium and acid–acetate methods, show a spectral shape similar to that of brushite. The spectra corresponding to the HPO_4_^2−^ group occur at wavenumbers of 1100 cm^−1^ and 1187 cm^−1^, while the signals, i.e., 575 cm^−1^, 977 cm^−1^, and 1039 cm^−1^, correspond to the PO_4_^3−^ group.

A signal was observed at about 1635 cm^−1^, particularly evident for ceramic powders with the lowest Ca/P ratio produced using either method. This signal originates from OH^−^ and is related to the bending vibrations of water molecules (δ(H_2_O)). The stretching vibration (ν(OH)), in turn, is characterized by a broad band in the range 3100 cm^−1^–3500 cm^−1^ [42,43,44,45]. The spectra for ceramic powders with higher Ca/P ratios show a very similar shape. Powders with number 3 presented spectra characteristic of hydroxyapatite. They show particularly high bands at wave numbers 560 cm^−1^, 600 cm^−1^ and 1040 cm^−1^ corresponding to the PO_4_^3−^ group, which is particularly characteristic of HAp [46,47].

FT-IR analysis confirmed that calcium phosphate ceramics were successfully obtained via three different syntheses. Analysis of the spectra showed a difference between the ceramic powders obtained within a single synthesis, due to the use of different Ca/P ratios.

### 3.3. X-Ray Diffraction Analysis

**Figure 2:**
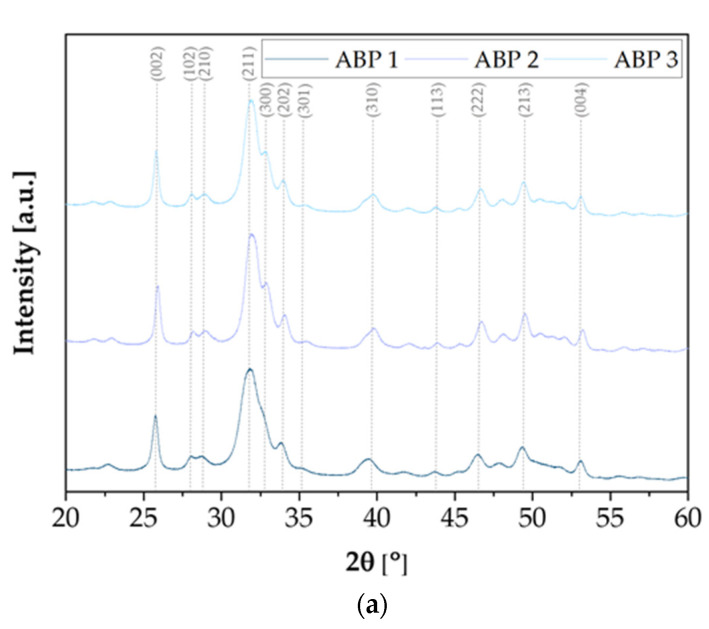

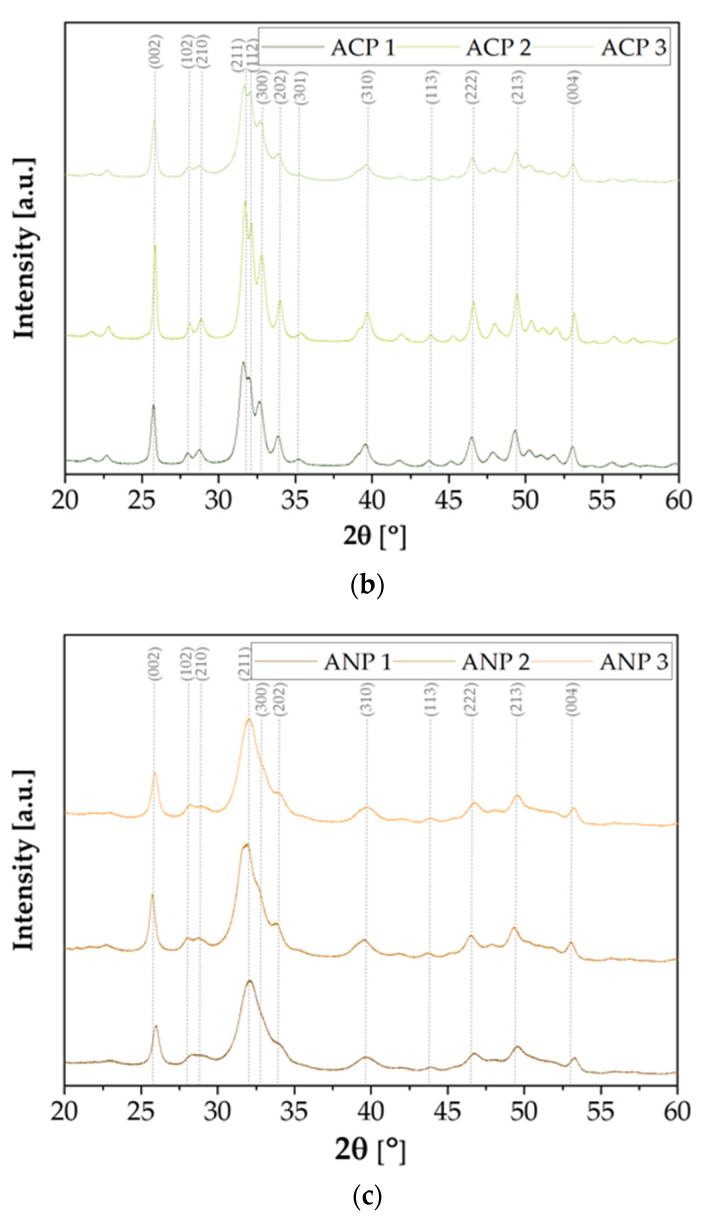
Diffractogram of the obtained powders by method: (**a**) ABP; (**b**) ACP; (**c**) ANP.

X-ray diffraction analysis was carried out to study the crystal structure of the synthesized calcium phosphate powders. The diffractograms shown in Figure 2 present the results of the X-ray analysis of ceramic powders obtained using three different methods: ABP, ACP, and ANP. In all cases, characteristic diffraction peaks are visible, confirming the presence of a crystalline phase corresponding to hydroxyapatite with a hexagonal structure (ICDD 01-080-7085, spatial arrangement P6_3_/m). The XRD spectra of the samples obtained using each method are similar to each other, regardless of the assumed Ca/P molar ratio. The most intense peak, observed at a 2θ angle of around 31.74°, corresponds to the main plane reflection (211) of hydroxyapatite. Samples obtained using the ABP and ACP methods are characterized by clear, sharp peaks of high intensity, indicating the high degree of crystallinity of these powders. Samples obtained using the ACP method additionally show a characteristic peak at a 2θ angle of 32.12°, corresponding to reflection (112). In addition, a peak at 33.92° 2θ, associated with the reflection (301), is present in both ACP and ABP samples, while this peak is absent in the powders obtained using the ANP method. The ANP method is also characterized by slightly lower signal intensity, which may indicate a lower degree of crystallinity or differences in crystallite size. Despite the observed differences, all of the samples obtained clearly exhibit the hexagonal structure of hydroxyapatite, regardless of the synthesis method used.

### 3.4. Particle Size Analysis

**Figure 3:**
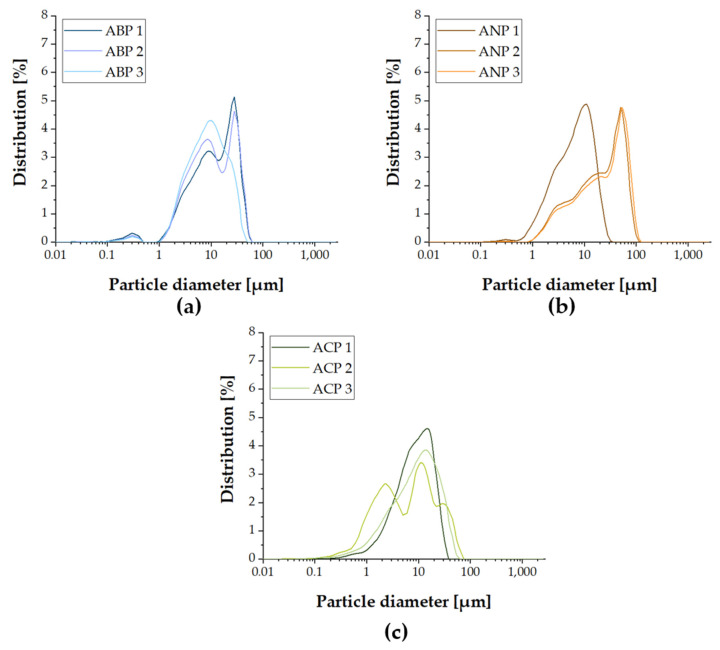
Histogram of particle size distribution for ceramic powders based on (**a**) ABP; (**b**) ANP; and (**c**) ACP.

Analysis of particle sizes obtained using the ABP, ANP, and ACP methods presented significant differences in their distribution and average values (Figure 3). Samples obtained using the ANP method are characterized by the largest values of the parameters *D*_10_, *D*_50_, and *D*_90_ (Table 2), indicating the dominance of larger particles, with average particle sizes ranging from 7.96 µm (ANP 1) to 32.12 µm (ANP 3). In the case of samples produced using the ABP method, smaller values of average particle size (11.42–15.38 µm) were observed, suggesting a more effective control over particle fragmentation. In contrast, samples obtained using the ACP method show the greatest heterogeneity, with average particle sizes ranging from 10.29 µm (ACP 1) to 31.387 µm (ACP 2), with the extremely high D_90_ value for ACP 2 (114.946 µm) indicating the presence of large agglomerates. These results suggest that the ANP method favors larger particles, and the ABP method produces more homogeneous and finer particles. In contrast, the ACP method may lead to uncontrolled agglomeration, requiring further optimization of synthesis conditions.

### 3.5. Morphology Analysis

**Figure 4:**
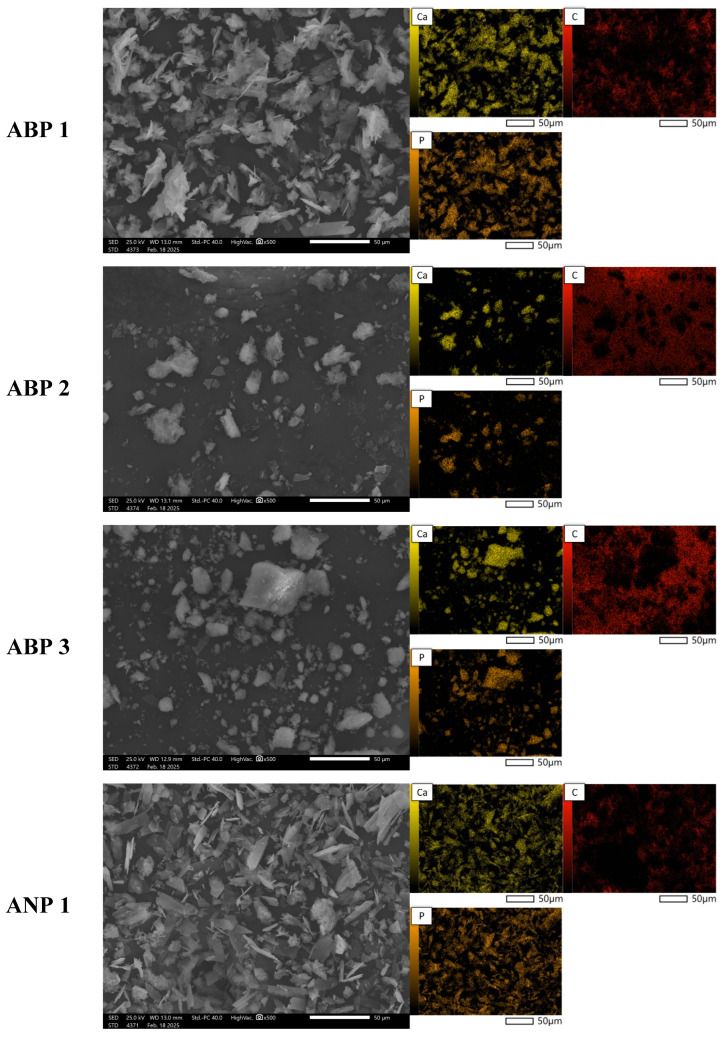

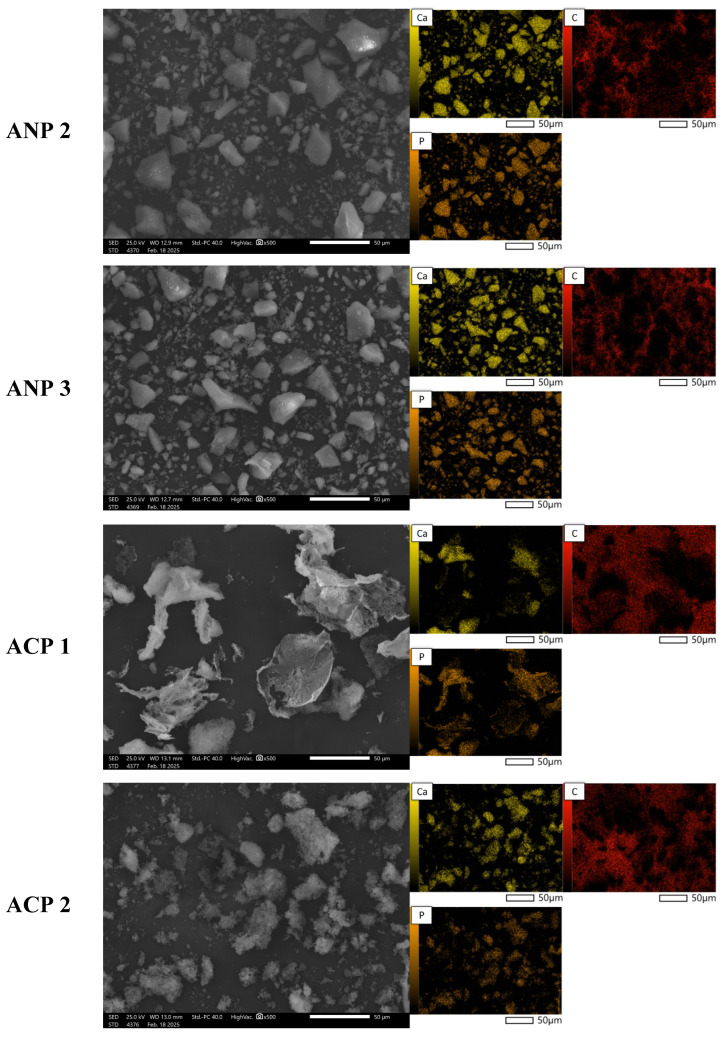

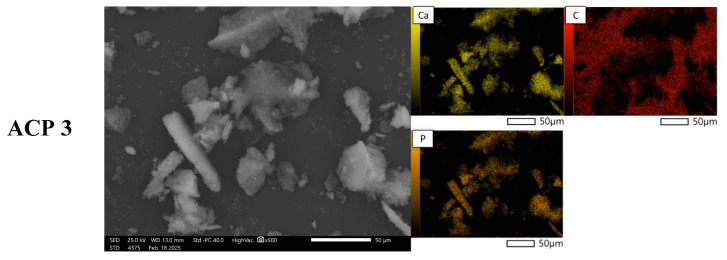
SEM images with elemental mapping for the obtained calcium phosphate powders.

Figure 4 presents SEM images of powder grains at 500× magnification, along with the elemental distribution maps for calcium, phosphorus, and carbon. The materials obtained using the three methods differ significantly in both morphology and chemical composition. SEM analysis reveals that the ANP method leads to large agglomerates with irregular shapes (ANP 2 and ANP 3), while ANP 1 and ABP 1 exhibit a structure with sharp edges and clearly defined ends. In contrast, ABP 2 and ABP 3 powders are characterized by irregularly shaped finer agglomerates. Materials obtained using the ACP method show asymmetric forms resembling cauliflower-like structures, which may be due to a different mechanism of particle growth.

The elemental composition of the synthesized calcium phosphate powders obtained was determined using EDS microanalysis. The results of the analysis are expressed as mass % (%M.) and atomic % (%At.) and are shown in Table 3. The table presents the content of elements detected on the studied surface via microscopic analysis. The presence of carbon is due to the carbon pads on which the ceramic powders were placed.

Analysis of the elemental composition by means of SEM-EDS confirms the presence of calcium-phosphate phases, as indicated by the overlap between calcium and phosphorus signals in the chemical composition maps. The results indicate that the content of these elements is highest in samples obtained using the ammonium method, especially in ANP 3, where the calcium concentration is 8.38%M (3.09%At.), suggesting more efficient mineralization of the material. ACP samples are characterized by intermediate values, while materials obtained using the ABP method show the lowest phosphorus and calcium contents, indicating that they are less saturated with mineral phases. The results suggest that the ammonium (ANP) method is the most favorable in terms of synthesizing phosphate–calcium ceramics, making it a promising approach for producing bioactive materials with potential biomedical applications.

### 3.6. Cytocompatibility

Cytocompatibility represents a fundamental prerequisite for composites intended for targeted bone tissue regeneration. By providing adequate biocompatibility at the cellular level, biomaterials such as ceramic powders support cell adhesion, migration, osteogenic differentiation, and osteoblast maturation, thereby promoting effective and stable osseointegration [48,49].

In this study, we assessed the cytocompatibility of three types of ceramic powders (ABP, ANP, and ACP) by measuring their metabolic activity using the MTT reduction assay, in accordance with ISO 10993-5:2009. To better reflect the potential application of the tested ceramic powders in bone tissue regeneration, cytocompatibility was assessed not only using the reference murine L929 fibroblast cell line but also human hFOB 1.19 osteoblasts.

**Figure 5:**
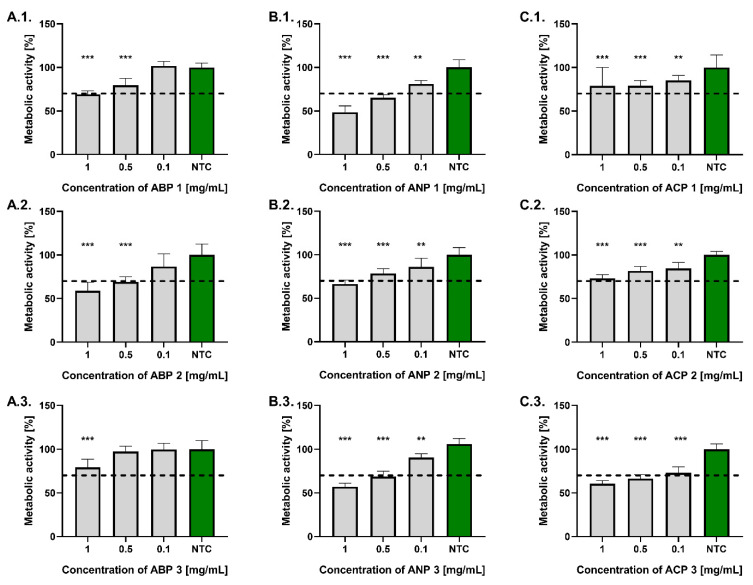
The metabolic activity of reference mouse L929 fibroblasts exposed for 24 h to ABP (**A.1.**–**A.3.**), ANP (**B.1.**–**B.3.**), and ACP (**C.1.**–**C.3.**). Cells incubated in the cell culture medium alone served as a non-treated control (NTC) of cells’ metabolic activity (100%). Data are presented as the mean ± standard deviation (SD) of five separate experiments (six replicates for each experimental variant). The black dashed line indicates the minimum level (70%) of fibroblasts’ metabolic activity, according to the ISO norm. * *p* < 0.05; ** *p* < 0.01; *** *p* < 0.001, statistically significant differences.

As shown in Figure 5, all tested powders at a concentration of 0.1 mg/mL complied with the ISO 10993-5:2009 standard, maintaining L929 fibroblast metabolic activity above 70% relative to untreated controls. At a concentration of 0.5 mg/mL, significant differences in fibroblast viability were observed among the tested powders. Compliance with the ISO standard was demonstrated by ABP 1, ABP 2, ANP 2, ACP 1, and ACP 2. The highest metabolic activity at a concentration of 0.5 mg/mL was observed in fibroblasts treated with ABP 3 (97.5 ± 6.3%), whereas the lowest was recorded for those exposed to ANP 1 (65.5 ± 3.3%). At a concentration of 1 mg/mL, only ACP 1, ACP 2, and ABP 3 met the requirements of the ISO standard. The highest metabolic activity was found in cells treated with ABP 1 (79.5 ± 8.0%), whereas the lowest was recorded following exposure to ANP 1 (48.7 ± 7.2%).

**Figure 6:**
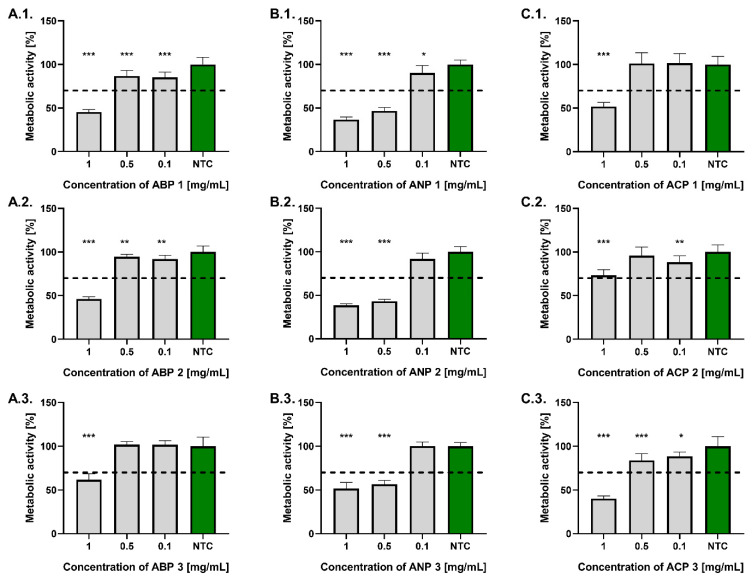
The metabolic activity of human hFOB 1.19 osteoblasts exposed for 24 h to ABP (**A.1.**–**A.3.**), ANP (**B.1**.–**B.3.**), and ACP (**C.1.**–**C.3.**). Cells incubated in the cell culture medium alone served as a non-treated control (NTC) of cells’ metabolic activity (100%). Data are presented as the mean ± standard deviation (SD) of five separate experiments (six replicates for each experimental variant). The black dashed line indicates the minimum level (70%) of osteoblasts’ metabolic activity, according to the ISO norm. * *p* < 0.05; ** *p* < 0.01; *** *p* < 0.001, statistically significant differences.

Similarly to the results obtained with the L929 fibroblast model, none of the tested ceramic powders at a concentration of 0.1 mg/mL reduced the metabolic activity of hFOB 1.19 osteoblasts by more than 30% compared to the untreated control (Figure 6). All ceramic powders from the ABP and ACP series, at a concentration of 0.5 mg/mL, also complied with the ISO 10993-5:2009 standard. The highest metabolic activity was found in osteoblasts treated with ACP 1 (101.2 ± 12.4%), whereas the lowest was observed for those exposed to ANP 2 (43.2 ± 2.2%). At a concentration of 1 mg/mL, only ACP 2 met the ISO standard, where the osteoblast’s metabolic activity after treatment was 73.2 ± 6.6%, while the most cytotoxic to those cells was ANP 1 (36.7 ± 3.0%).

### 3.7. NF-κB Activation

**Figure 7:**
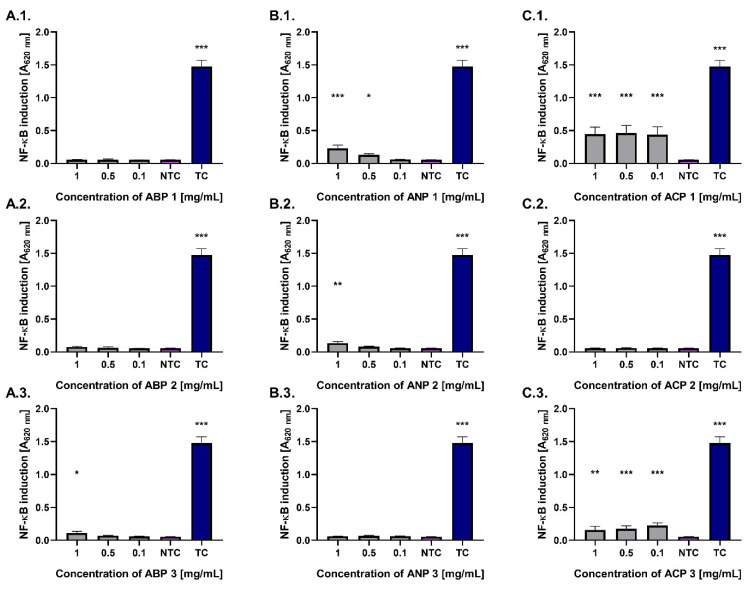
The level of activation of THP1-Blue™ NF-κB monocytes incubated for 24 h with ABP (**A.1.**–**A.3.**), ANP (**B.1.**–**B.3.**), and ACP (**C.1.**–**C.3.**). Monocytes in culture medium alone served as a non-treated control (NTC). LPS of Escherichia coli O55:B5 was used as a treated control (TC) of NF-κB induction. Data are presented as the mean ± standard deviation (SD) of five separate experiments (six replicates of each experimental variant). * *p* < 0.05; ** *p* < 0.01; *** *p* < 0.001, statistically significant differences. A (absorbance)—optical density 620 nm.

Activation of the NF-κB transcription factor underlies two key physiological processes: immune system activation and the initiation of tissue regeneration, including bones. In the present study, NF-κB activation was evaluated using a THP1-Blue™ NF-κB reporter monocyte model after 24 h of exposure to the tested ceramic powders (Figure 7).

Most of the tested ceramic powders did not activate the NF-κB pathway, showing stimulation levels comparable to the untreated control (0.06). However, monocytes treated with ACP 1 and ACP 3 at a concentration of 0.1 mg/mL exhibited statistically significant increases in NF-κB pathway activation (*p* < 0.001), with values of 0.44 ± 0.12 and 0.23 ± 0.04, respectively. Significant (*p* < 0.05) activation of the nuclear transcription factor NF-κB was also observed in the monocyte model for ANP 1 (0.13 ± 0.05) at a concentration of 0.5 mg/mL. The highest number of ceramics activating the studied pathway was identified at a concentration of 1 mg/mL, including ACP 1, ANP 1, ANP 2, and ACP 3. All tested ceramic powders exhibited significantly lower activation compared to the treated control (LPS).

## 4. Discussion and Conclusions

The present study compares the physicochemical and biological properties of calcium phosphate powders synthesized via three wet precipitation methods. The primary objective was to evaluate the impact of the synthesis method, processing conditions, and Ca/P molar ratio on their potential application in bone tissue engineering.

Chemical analysis revealed that modulation of the pH during synthesis and the adjustment of reactant proportions allowed for controlled variation of the Ca/P molar ratio, achieving values between 1.32 and 1.88. These results confirm that the powders differed significantly in composition, with the Ca/P ratio strongly influencing material properties; in particular, lower Ca/P ratios are associated with faster resorption rates in vivo [50]. FT-IR and XRD analyses corroborated the presence of different calcium-phosphate phases depending on the Ca/P ratio. Samples with lower Ca/P values exhibited characteristics of DCPD-like phases, while higher ratios were indicative of HAp formation. These findings are consistent with previous reports, where hydroxyapatite demonstrates distinctive FT-IR absorption bands at approximately 560, 600, and 1040 cm^−1^ [51]. Differences in particle size distribution were also observed among the synthesis methods. Powders synthesized via the ANP method, particularly those with Ca/P ratios of 1.63 and 1.88, exhibited the largest particle sizes. However, no clear correlation between synthesis method and particle size was established, likely due to the presence of agglomerates resistant to ultrasonic dispersion. SEM and EDS analyses further confirmed morphological and compositional differences among the synthesized powders. Notably, the ANP-derived materials, and particularly the ANP 3 sample, demonstrated higher calcium and phosphorus contents, indicating enhanced mineralization and suggesting improved bioactivity potential. This sample also displayed the highest measured Ca/P molar ratio. As the synthesized calcium phosphate materials were obtained in powder form, porosity parameters were not quantitatively determined. However, these powders are intended as precursors for composite, 3D-printed biomaterials, where the analysis of macro/microporosity will be of key importance.

Cytocompatibility is a fundamental requirement for new biomaterials intended for bone tissue regeneration, as it is crucial for proper and lasting osseointegration and for restoring the mechanical functionality of bone [52]. The absence of cytotoxicity is crucial for maintaining an appropriate rate of migration and adhesion of mesenchymal cells and osteoblasts, as well as for supporting their proliferation, differentiation, and maturation into osteocytes [53]. In this study, we demonstrated that all tested calcium phosphate powders were cytocompatible at a concentration of 0.1 mg/mL with L929 fibroblasts and hFOB 1.19 osteoblasts and did not reduce their metabolic activity below 70% compared to untreated cells, thus complying with the ISO 10993-5:2009 standard. Depending on the type of synthesized powder, variations in cellular metabolic activity were observed, which may be attributed to differences in morphology, calcium and phosphorus ion content, or the presence of residual, unwashed substrates.

Interactions between the immune system and bone cells play a key role in regulating the proper course of damaged bone tissue regeneration. One of the most important transcription factors involved in this process is NF-κB, whose activation is essential for the stimulation of immune cells, the initiation of bone regeneration, and the onset of bone remodeling through the targeted resorptive activity of osteoclasts [54,55,56]. While low immune cell activation, including NF-κB signaling, supports osseointegration, excessive activation can markedly hinder the healing of damaged bone tissue [54,55,57]. Thus, evaluating this parameter is of critical importance. Assessment of NF-κB pathway activation using a THP-1 monocyte reporter model revealed that only the ACP powders triggered its activation at cytocompatible concentrations, which may indicate a higher pro-regenerative potential. The low or absent activity of this pathway observed for all powders may suggest better biocompatibility, although further immunological studies are required.

In summary, the synthesized calcium phosphate powders exhibited a controllable composition, favorable cell compatibility, and distinct phase properties, which were influenced by the Ca/P molar ratio and the synthesis method. Due to its chemical stability, biocompatibility, and morphology, this ceramic shows great potential as a functional component or reinforcing phase in composite biomaterials, especially in 3D-printed scaffolds for bone tissue regeneration.

## Figures and Tables

**Table 1:** Ca/P ratio of produced ceramic powders.

Method	Sample	%Ca	%P	Ca/P	pH
ABP	ABP 1	33.53 ± 0.23	18.13 ± 0.94	1.43	~5/6
	ABP 2	42.35 ± 0.23	21.10 ± 0.40	1.55	~7
	ABP 3	43.82 ± 0.23	19.26 ± 0.28	1.76	~11
ANP	ANP 1	36.61 ± 0.23	21.43 ± 0.18	1.32	~5/6
	ANP 2	40.08 ± 0.40	19.00 ± 0.39	1.63	~7
	ANP 3	44.49 ± 0.40	18.28 ± 0.09	1.88	~11
ACP	ACP 1	36.21 ± 0.23	19.75 ± 0.21	1.42	~5/6
	ACP 2	40.35 ± 0.23	19.48 ± 0.25	1.60	~7
	ACP 3	44.89 ± 0.40	20.20 ± 0.22	1.72	~11

**Table 2:** Particle size average for the obtained ceramic powders.

Sample	D_10_ [µm]	D_50_ [µm]	D_90_ [µm]	Mean Size [µm]
**ABP 1**	2.423 ± 0.019	10.967 ± 0.301	31.749 ± 2.337	15.376 ± 0.860
**ABP 2**	2.576 ± 0.049	9.608 ± 0.291	32.636 ± 2.308	15.038 ± 0.747
**ABP 3**	2.466 ± 0.062	8.212 ± 0.537	23.943 ± 1.718	11.424 ± 0.817
**ANP 1**	1.684 ± 0.009	6.406 ± 0.066	15.258 ± 0.343	7.955 ± 0.131
**ANP 2**	3.066 ± 0.051	20.602 ± 0.725	59.210 ± 3.956	28.171 ± 1.378
**ANP 3**	3.331 ± 0.066	24.065 ± 1.023	66.988 ± 5.918	32.120 ± 2.128
**ACP 1**	2.279 ± 0.202	8.173 ± 0.433	19.749 ± 1.775	10.291 ± 0.801
**ACP 2**	1.019 ± 0.180	7.008 ± 3.671	114.946 ± 142.505	31.387 ± 32.679
**ACP 3**	1.646 ± 0.046	8.689 ± 0.550	26.843 ± 0.817	12.470 ± 0.416

**Table 3:** Elemental EDS analysis of the obtained ceramic powders.

Sample	C		O		P		Ca	
	%M.	%At.	%M.	%At.	%M.	%At.	%M.	%At.
**ABP 1**	40.92 ± 0.50	51.15 ± 0.62	46.27 ± 0.99	43.42 ± 0.93	5.70 ± 0.12	2.76 ± 0.06	7.11 ± 0.14	2.66 ± 0.05
**ABP 2**	66.03 ± 0.45	72.91 ± 0.50	31.66 ± 0.91	26.24 ± 0.75	0.85 ± 0.05	0.36 ± 0.02	1.46 ± 0.06	0.48 ± 0.02
**ABP 3**	56.02 ± 0.51	65.38 ± 0.59	35.98 ± 1.00	31.53 ± 0.87	2.82 ± 0.09	1.28 ± 0.04	5.18 ± 0.13	1.81 ± 0.04
**ANP 1**	41.66 ± 0.55	52.12 ± 0.69	44.88 ± 1.06	42.15 ± 1.00	6.21 ± 0.14	3.01 ± 0.07	7.25 ± 0.16	2.72 ± 0.06
**ANP 2**	46.19 ± 0.50	57.19 ± 0.61	39.92 ± 0.97	37.11 ± 0.90	5.09 ± 0.11	2.44 ± 0.05	8.80 ± 0.15	3.26 ± 0.06
**ANP 3**	47.47 ± 0.49	58.41 ± 0.60	39.02 ± 0.94	36.05 ± 0.87	5.12 ± 0.11	2.44 ± 0.05	8.38 ± 0.15	3.09 ± 0.05
**ACP 1**	56.31 ± 0.48	65.29 ± 0.56	35.28 ± 0.86	30.71 ± 0.75	2.05 ± 0.07	0.92 ± 0.03	1.67 ± 0.07	0.58 ± 0.02
**ACP 2**	59.17 ± 0.51	67.51 ± 0.58	35.37 ± 0.99	30.29 ± 0.85	1.95 ± 0.08	0.86 ± 0.03	2.98 ± 0.10	1.02 ± 0.03
**ACP 3**	58.03 ± 0.52	66.92 ± 0.60	35.21 ± 1.02	30.48 ± 0.88	2.55 ± 0.09	1.14 ± 0.04	4.21 ± 0.12	1.46 ± 0.04

## Data Availability

The data presented in this study are openly available in Mendeley Data at 10.17632/my5v9ygg7d.1.

## References

[1] Hou X., Zhang L., Zhou Z., Luo X., Wang T., Zhao X., Lu B., Chen F., Zheng L. (2022). Calcium Phosphate-Based Biomaterials for Bone Repair. J. Funct. Biomater..

[2] Yuan H., Yang Z., Li Y., Zhang X., De Bruijn J.D., De Groot K. (1998). Osteoinduction by Calcium Phosphate Biomaterials. J. Mater. Sci. Mater. Med..

[3] Calafiori A.R., Di Marco G., Martino G., Marotta M. (2007). Preparation and Characterization of Calcium Phosphate Biomaterials. J. Mater. Sci. Mater. Med..

[4] Lee J.S., Murphy W.L. (2013). Functionalizing Calcium Phosphate Biomaterials with Antibacterial Silver Particles. Adv. Mater..

[5] Verron E., Khairoun I., Guicheux J., Bouler J.M. (2010). Calcium Phosphate Biomaterials as Bone Drug Delivery Systems: A Review. Drug Discov. Today.

[6] Roycroft P.D., Cuypers M. (2015). The Etymology of The Mineral Name ‘Apatite’: A Clarification. Ir. J. Earth Sci..

[7] Jeong J., Kim J.H., Shim J.H., Hwang N.S., Heo C.Y. (2019). Bioactive Calcium Phosphate Materials and Applications in Bone Regeneration. Biomater. Res..

[8] Helmus M.N., Gibbons D.F., Cebon D. (2008). Biocompatibility: Meeting a Key Functional Requirement of Next-Generation Medical Devices. Toxicol. Pathol..

[9] Sasikumar Y., Rajendran N. (2012). Surface Modification and In Vitro Characterization of Cp-Ti and Ti-5Al-2Nb-1Ta Alloy in Simulated Body Fluid. J. Mater. Eng. Perform..

[10] Bansiddhi A., Dunand D.C. (2011). Processing of NiTi Foams by Transient Liquid Phase Sintering. J. Mater. Eng. Perform..

[11] Gross K.A., Muller D., Lucas H., Haynes D.R. (2012). Osteoclast Resorption of Thermal Spray Hydoxyapatite Coatings Is Influenced by Surface Topography. Acta Biomater..

[12] Puckett S.D., Lee P.P., Ciombor D.M., Aaron R.K., Webster T.J. (2010). Nanotextured Titanium Surfaces for Enhancing Skin Growth on Transcutaneous Osseointegrated Devices. Acta Biomater..

[13] Kärrholm J., Razaznejad R. (2008). Fixation and Bone Remodeling Around a Low Stiffness Stem in Revision Surgery. Clin. Orthop. Relat. Res.®.

[14] Nomura N., Sakamoto K., Takahashi K., Kato S., Abe Y., Doi H., Tsutsumi Y., Kobayashi M., Kobayashi E., Kim W.-J. (2010). Fabrication and Mechanical Properties of Porous Ti/HA Composites for Bone Fixation Devices. Mater. Trans..

[15] Arifin A., Sulong A.B., Muhamad N., Syarif J., Ramli M.I. (2014). Material Processing of Hydroxyapatite and Titanium Alloy (HA/Ti) Composite as Implant Materials Using Powder Metallurgy: A Review. Mater. Des..

[16] Nath S., Kalmodia S., Basu B. (2010). Densification, Phase Stability and in Vitro Biocompatibility Property of Hydroxyapatite-10 Wt% Silver Composites. J. Mater. Sci. Mater. Med..

[17] Sommerfeldt D., Rubin C. (2001). Biology of Bone and How It Orchestrates the Form and Function of the Skeleton. Eur. Spine J..

[18] Dorozhkin S.V., Epple M. (2002). Biological and Medical Significance of Calcium Phosphates. Angew. Chem. Int. Ed..

[19] Eliaz N., Metoki N. (2017). Calcium Phosphate Bioceramics: A Review of Their History, Structure, Properties, Coating Technologies and Biomedical Applications. Materials.

[20] Khalid H., Chaudhry A.A., Khan A.S., Chaudhry A.A. (2020). 4—Basics of Hydroxyapatite—Structure, Synthesis, Properties, and Clinical Applications. Handbook of Ionic Substituted Hydroxyapatites.

[21] Ching W.Y., Rulis P., Misra A. (2009). Ab Initio Elastic Properties and Tensile Strength of Crystalline Hydroxyapatite. Acta Biomater..

[22] Guo X., Lei L., Xiao H., Zheng J. (2020). Effect of Remineralisation on the Mechanical Properties and Tribological Behaviour of Human Tooth Dentine. Biosurface Biotribology.

[23] He M., Hou Y., Zhu C., He M., Jiang Y., Feng G., Liu L., Li Y., Chen C., Zhang L. (2021). 3D-Printing Biodegradable PU/PAAM/Gel Hydrogel Scaffold with High Flexibility and Self-Adaptibility to Irregular Defects for Nonload-Bearing Bone Regeneration. Bioconjugate Chem..

[24] Toledano M., Vallecillo-Rivas M., Osorio M.T., Muñoz-Soto E., Toledano-Osorio M., Vallecillo C., Toledano R., Lynch C.D., Serrera-Figallo M.-A., Osorio R. (2021). Zn-Containing Membranes for Guided Bone Regeneration in Dentistry. Polymers.

[25] Samavedi S., Whittington A.R., Goldstein A.S. (2013). Calcium Phosphate Ceramics in Bone Tissue Engineering: A Review of Properties and Their Influence on Cell Behavior. Acta Biomater..

[26] Ogata K., Imazato S., Ehara A., Ebisu S., Kinomoto Y., Nakano T., Umakoshi Y. (2005). Comparison of Osteoblast Responses to Hydroxyapatite and Hydroxyapatite/Soluble Calcium Phosphate Composites. J. Biomed. Mater. Res. Part A.

[27] Bohner M., Santoni B.L.G., Döbelin N. (2020). β-Tricalcium Phosphate for Bone Substitution: Synthesis and Properties. Acta Biomater..

[28] Dorozhkin S.V. (2009). Calcium Orthophosphates in Nature, Biology and Medicine. Materials.

[29] Owen G.R., Dard M., Larjava H. (2018). Hydoxyapatite/Beta-Tricalcium Phosphate Biphasic Ceramics as Regenerative Material for the Repair of Complex Bone Defects. J. Biomed. Mater. Res. Part B Appl. Biomater..

[30] Bi L., Cheng W., Fan H., Pei G. (2010). Reconstruction of Goat Tibial Defects Using an Injectable Tricalcium Phosphate/Chitosan in Combination with Autologous Platelet-Rich Plasma. Biomaterials.

[31] Combes C., Rey C. (2010). Amorphous Calcium Phosphates: Synthesis, Properties and Uses in Biomaterials. Acta Biomater..

[32] Popp J.R., Laflin K.E., Love B.J., Goldstein A.S. (2011). In Vitro Evaluation of Osteoblastic Differentiation on Amorphous Calcium Phosphate-Decorated Poly(Lactic-Co-Glycolic Acid) Scaffolds. J. Tissue Eng. Regen. Med..

[33] (1997). Pasze—Fosforany Paszowe.

[34] (1988). Nawozy Sztuczne.

[35] Słota D., Urbaniak M.M., Tomaszewska A., Niziołek K., Włodarczyk M., Florkiewicz W., Szwed-Georgiou A., Krupa A., Sobczak-Kupiec A. (2024). Crosslinked Hybrid Polymer/Ceramic Composite Coatings for the Controlled Release of Clindamycin. Biomater. Sci..

[36] Urbaniak M.M., Gazińska M., Rudnicka K., Płociński P., Nowak M., Chmiela M. (2023). In Vitro and In Vivo Biocompatibility of Natural and Synthetic *Pseudomonas aeruginosa* Pyomelanin for Potential Biomedical Applications. Int. J. Mol. Sci..

[37] Urbaniak M.M., Rudnicka K., Płociński P., Chmiela M. (2024). Exploring the Osteoinductive Potential of Bacterial Pyomelanin Derived from Pseudomonas Aeruginosa in a Human Osteoblast Model. Int. J. Mol. Sci..

[38] (2009). Biological Evaluation of Medical Devices-Part 5: Tests for In Vitro Cytotoxicity.

[39] Kurzyk A., Szwed-Georgiou A., Pagacz J., Antosik A., Tymowicz-Grzyb P., Gerle A., Szterner P., Włodarczyk M., Płociński P., Urbaniak M.M. (2023). Calcination and Ion Substitution Improve Physicochemical and Biological Properties of Nanohydroxyapatite for Bone Tissue Engineering Applications. Sci. Rep..

[40] Mohammadi M., Tulliani J.-M., Montanaro L., Palmero P. (2021). Gelcasting and Sintering of Hydroxyapatite Materials: Effect of Particle Size and Ca/P Ratio on Microstructural, Mechanical and Biological Properties. J. Eur. Ceram. Soc..

[41] Brazete D., Torres P.M.C., Abrantes J.C.C., Ferreira J.M.F. (2018). Influence of the Ca/P Ratio and Cooling Rate on the Allotropic A↔β-Tricalcium Phosphate Phase Transformations. Ceram. Int..

[42] Niziołek K., Słota D., Ronowska A., Sobczak-Kupiec A. (2025). Calcium Phosphate Biomaterials Modified with Mg^2+^ or Mn^2+^ Ions: Structural, Chemical, and Biological Characterization. Ceram. Int..

[43] Binitha M.P., Pradyumnan P.P. (2013). Dielectric Property Studies of Biologically Compatible Brushite Single Crystals Used as Bone Graft Substitute. J. Biomater. Nanobiotechnology.

[44] Laskus-Zakrzewska A., Zgadzaj A., Kolmas J. (2021). Synthesis and Physicochemical Characterization of Zn-Doped Brushite. Ceram. Int..

[45] Idowu B., Cama G., Deb S., Di Silvio L. (2014). In Vitro Osteoinductive Potential of Porous Monetite for Bone Tissue Engineering. J. Tissue Eng..

[46] Liu Y., Sebastian S., Huang J., Corbascio T., Engellau J., Lidgren L., Tägil M., Raina D.B. (2022). Longitudinal in Vivo Biodistribution of Nano and Micro Sized Hydroxyapatite Particles Implanted in a Bone Defect. Front. Bioeng. Biotechnol..

[47] Sadlik J., Kosińska E., Bańkosz M., Tomala A., Bruzda G., Jampilek J., Sobczak-Kupiec A. (2024). Gradient Titanium Alloy with Bioactive Hydroxyapatite Porous Structures for Potential Biomedical Applications. Materials.

[48] Schafer S., Al-Qaddo H., Gosau M., Smeets R., Hartjen P., Friedrich R.E., Nada O.A., Vollkommer T., Rashad A. (2021). Cytocompatibility of Bone Substitute Materials and Membranes. In Vivo.

[49] Wong S.K., Yee M.M.F., Chin K.-Y., Ima-Nirwana S. (2023). A Review of the Application of Natural and Synthetic Scaffolds in Bone Regeneration. J. Funct. Biomater..

[50] Dorozhkin S.V. (2015). Calcium Orthophosphates (CaPO4): Occurrence and Properties. Progress Biomater..

[51] Friederichs R.J., Chappell H.F., Shepherd D.V., Best S.M. (2015). Synthesis, Characterization and Modelling of Zinc and Silicate Co-Substituted Hydroxyapatite. J. R. Soc. Interface.

[52] Gutiérrez-Prieto S.J., Perdomo-Lara S.J., Diaz-Peraza J.M., Sequeda-Castañeda L.G. (2019). Analysis of In Vitro Osteoblast Culture on Scaffolds for Future Bone Regeneration Purposes in Dentistry. Adv. Pharmacol. Sci..

[53] Xue N., Ding X., Huang R., Jiang R., Huang H., Pan X., Min W., Chen J., Duan J.-A., Liu P. (2022). Bone Tissue Engineering in the Treatment of Bone Defects. Pharmaceuticals.

[54] Jimi E., Takakura N., Hiura F., Nakamura I., Hirata-Tsuchiya S. (2019). The Role of NF-κB in Physiological Bone Development and Inflammatory Bone Diseases: Is NF-κB Inhibition “Killing Two Birds with One Stone”?. Cells.

[55] Lopez E.M., Leclerc K., Ramsukh M., Parente P.E., Patel K., Aranda C.J., Josephson A.M., Remark L.H., Kirby D.J., Buchalter D.B. (2022). Modulating the Systemic and Local Adaptive Immune Response after Fracture Improves Bone Regeneration during Aging. Bone.

[56] Boyce B.F., Li J., Yao Z., Xing L. (2023). Nuclear Factor-Kappa B Regulation of Osteoclastogenesis and Osteoblastogenesis. Endocrinol. Metab..

[57] Baht G.S., Vi L., Alman B.A. (2018). The Role of the Immune Cells in Fracture Healing. Curr. Osteoporos. Rep..

